# Integrative Analysis of Multi-Omics Identified the Prognostic Biomarkers in Acute Myelogenous Leukemia

**DOI:** 10.3389/fonc.2020.591937

**Published:** 2020-12-10

**Authors:** Jiafeng Zheng, Tongqiang Zhang, Wei Guo, Caili Zhou, Xiaojian Cui, Long Gao, Chunquan Cai, Yongsheng Xu

**Affiliations:** ^1^ Department of Pediatric Respiratory Medicine, Tianjin Children’s Hospital (Tianjin University Children’s Hospital), Tianjin, China; ^2^ Department of Science and Education, Tianjin Children’s Hospital (Tianjin University Children’s Hospital), Tianjin, China; ^3^ Department of Clinical Lab, Tianjin Children’s Hospital (Tianjin University Children’s Hospital), Tianjin, China; ^4^ Department of Pediatric Endocrinology, Tianjin Children’s Hospital (Tianjin University Children’s Hospital), Tianjin, China; ^5^ Tianjin Institute of Pediatrics (Tianjin Key Laboratory of Birth Defects for Prevention and Treatment), Tianjin Children’s Hospital (Tianjin University Children’s Hospital), Tianjin, China

**Keywords:** multi-omics, prognostic, acute myelogenous leukemia, children, methylation

## Abstract

**Background:**

Acute myelogenous leukemia (AML) is a common pediatric malignancy in children younger than 15 years old. Although the overall survival (OS) has been improved in recent years, the mechanisms of AML remain largely unknown. Hence, the purpose of this study is to explore the differentially methylated genes and to investigate the underlying mechanism in AML initiation and progression based on the bioinformatic analysis.

**Methods:**

Methylation array data and gene expression data were obtained from TARGET Data Matrix. The consensus clustering analysis was performed using ConsensusClusterPlus R package. The global DNA methylation was analyzed using methylationArrayAnalysis R package and differentially methylated genes (DMGs), and differentially expressed genes (DEGs) were identified using Limma R package. Besides, the biological function was analyzed using clusterProfiler R package. The correlation between DMGs and DEGs was determined using psych R package. Moreover, the correlation between DMGs and AML was assessed using varElect online tool. And the overall survival and progression-free survival were analyzed using survival R package.

**Results:**

All AML samples in this study were divided into three clusters at k = 3. Based on consensus clustering, we identified 1,146 CpGs, including 40 hypermethylated and 1,106 hypomethylated CpGs in AML. Besides, a total 529 DEGs were identified, including 270 upregulated and 259 downregulated DEGs in AML. The function analysis showed that DEGs significantly enriched in AML related biological process. Moreover, the correlation between DMGs and DEGs indicated that seven DMGs directly interacted with AML. CD34, HOXA7, and CD96 showed the strongest correlation with AML. Further, we explored three CpG sites cg03583857, cg26511321, cg04039397 of CD34, HOXA7, and CD96 which acted as the clinical prognostic biomarkers.

**Conclusion:**

Our study identified three novel methylated genes in AML and also explored the mechanism of methylated genes in AML. Our finding may provide novel potential prognostic markers for AML.

## Introduction

Acute myelogenous leukemia (AML) is a common pediatric hematologic cancer in children younger than 15 years (1, 2). In the past several years, the survival rate for AML has increased because of the improvement of therapeutic strategies, risk stratification, and supportive care (3, 4). It has been reported that the long-term survival rate of AML is about 70% in children (5). Although the antitumor treatment for AML exhibits the optimistic outcome, the initiation and development of AML remain unclear.

Recent researches have demonstrated epigenetic modifications act a critical role in AML through altering the transcriptional activation of oncogenes or tumor suppressors (6–8). DNA methylation is a common epigenetic modification in biological process and pathological processes in multiple tumors, such as regulation of tumorigenesis and development in breast cancer and lung cancer (9, 10). Previous study has found that there is abnormal DNA methylation before cancer cell mutation (11). In an experiment on breast cancer cells, some scholars have found that inhibition of DNA methylation can accelerate the spread and metastasis of breast cancer cells (12). Some scholars have also found that the changes of DNA methylation and transcriptome are related to the sensitivity of chemotherapeutic drugs (13, 14). Furthermore, scholars have found that cisplatin resistance is related to the hypermethylation deletion of several CpG sites mainly located in the intergenomic region, which directly affects the efficacy of treatment, thus affecting the survival time of patients (15). Moreover, increasing evidence has demonstrated that DNA methylated genes might act as the biomarkers in AML (16, 17). Multi-omics of AML has indicated that high frequent methylation events occurs in children than adults (18). Besides, it has been revealed that hypermethylation of the ASNS promoter inhibits ASNS expression to increase sensitive to L-asparaginase and Decitabine (19). Furthermore, previous studies found that azacitidine and decitabine act as the inhibitors of DNA methylation and have been used in chemotherapy in patients with AML (20, 21). Inhibiting the expression of DNA methylation can effectively improve the effect of chemotherapy and prolong the survival time of AML patients (22). Previous studies have found that DNA methylation is a high sensitivity and specificity biomarker of AML in distinguishing it from other leukemia (9). Besides DNA methylation being strongly associated with prognosis of AML patients, it has been reported that different methylation types have different clinical outcomes for AML patients (23). And DNA methylated genes can be used as therapeutic targets in AML (24).

Although numerous studies have illustrated the regulatory effects of DNA methylation in AML, research for prognostic biomarkers based on DNA methylated genes is still lacking. Hence, we conduct an integrative analysis of methylation and transcription in AML based on bioinformatics and identify differentially methylated genes in AML. Furthermore, we analyzed the correlation between differentially methylated genes and AM and explored the DNA methylation-related prognostic biomarkers for AML patients.

## Methods

### Data Collecting and Processing

DNA methylation data and gene expression profilers were obtained from 219 paired AML samples and normal samples in TARGET Data Matrix. DNA methylation data was carried out with Illumina HumanMethylation27K array, and gene expression profiles were carried out with Affymetrix Gene ST. The methylation levels were assessed using beta (*β*)-value which ranged from 0 to 1. *β*-value represented the ratio of the methylated allele frequency and unmethylated allele frequency. And the expression microarray data was normalized using quantile normalization methods.

### Unsupervised Consensus Clustering Analysis

The ConsensusClusterPlus R package was used for unsupervised consensus analysis, and the clustering was performed using pam method with sampling proportion 0.8. Importantly, the consensus clustering included the following parts. The consistent matrix (CM) plots were illustrated based on k-value. And empirical cumulative distribution function (CDF) plots revealed the consensus distributions for each k. The k at approximate maximum distribution indicated maximum stability cluster structure. The delta area plot showed the delta area score (y-axis); the delta area score determines the stability of clustering. Moreover, the item tracking plot exhibited the consensus cluster of items (columns) at each k (rows) to identify the stability of clustering. Cluster-consensus plot showed the cluster-consensus value at different k values; high cluster-consensus value indicated low stability of clustering. Item-consensus plot is the average consensus value between an item and members of a consensus cluster. An item corresponded to multiple item-consensus values with different ks.

### Differential DNA Methylation Analysis

The principal component analysis (PCA) was used to analysis the sample bias in this study. And then, DNA methylation analysis was performed using methylationArrayAnalysis R package. The probes containing single nucleotide polymorphisms (SNPs), probes located in chromosome X, and more than 10% missing values in the probes were excluded in this study. The differentially DNA methylation probes (DMPs) were predicated with a cutoff value |Δ*β*| ≥0.2 and adjusted P-value <0.05. And DNA methylation regions (DMRs) were analyzed with a cutoff value no. CpGs ≥3, |meandiff| ≥0.2, and fisher (P) >0.05.

### Differentially Expressed Gene Analysis

Limma R package was used to determine the DEGs between cluster 2 and cluster 3 in AML. The cutoff value was |log2 (fold change) | ≥ 1 and P-value < 0.05. The volcano plot of DEGs was drawn using ggplot R package, and heatmap of DEGs was drawn using pheatmap R package.

### Gene Ontology Annotation and Kyoto Encyclopedia of Genes and Genomes Enrichment Analysis

The biological function was analyzed by GO and KEGG pathway enrichment analysis using clusterProfiler R package with a threshold P-value <0.05. GO annotation included biological process (BP), molecular function (MF), and cellular component (CC).

### Correlation Between DMGs and DEGs

Correlation between DMGs and DEGs was calculated by Pearson correlation analysis using psych R package with a cutoff value correlation ≥0.5 and adjusted P-value <0.05. Moreover, the protein–protein interaction (PPI) network of hypomethylated-high expression genes and hypermethylated-low expression genes was constructed and visualized using Cytoscape.

### Correlation Between DMGs and AML

The correlation between DMGs and AML was analyzed using varElect online tool, and high score indicated the strong correlation.

### Survival Analysis

The overall survival (OS) and progression-free survival (PFS) were analyzed by Kaplan–Meier (KM) survival plots using survival R package based on *β*-value.

## Results

### Construction of Three Unsupervised Clusters in AML

**Figure 1:**
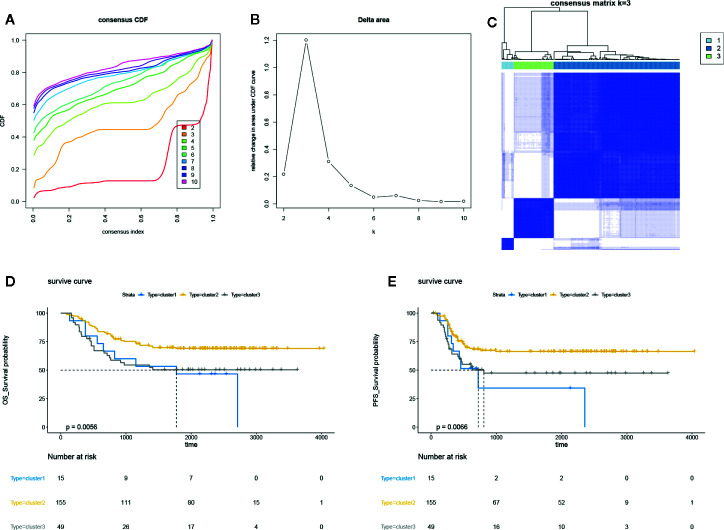
Unsupervised clustering analysis in AML. **(A)** The empirical cumulative distribution function (CDF) plots revealed the consensus distributions for each k. **(B)** The delta area score displayed the relative growth in cluster stability. **(C)** The circular manhattan (CM) plot exhibited the clusters at k = 3. **(D)** The overall survival (OS) plot for the AML patients of clusters 1, 2, 3 using survival R package. **(B)** progression-free survival (PFS) plot for the AML patients of clusters 1, 2, 3 using survival R package.

The DNA methylation array data of 219 AML samples was obtained from TARGET Data Matrix and normalized using exclusion criteria. Then, the consensus clustering was performed using ConsensusClusterPlus R package; the CDF showed the lowest rangeability at consensus index 0.2–0.6 with k = 3 (**Figure 1A**). The delta area scores 2.5 was the highest at k = 3 (**Figure 1B**). Besides, the CM plot also showed the highest consistency at k = 3 (**Figure 1C**, **Supplementary Figure 1A**). Besides, the results of the tracking plot (**Supplementary Figure 1B**), cluster consensus plot (**Supplementary Figure 1C**), item consensus plot (**Supplementary Figure 1D**) also indicated the cluster stability at k = 3. Thus, consensus clustering of AML samples identified three clusters, and the clusters 2 and 3 showed the maximum sample number could be used for subsequent analysis. We further assess the correlation between three clusters and survival rates; we found the differential DNA methylation has strong correlation with OS and PFS of patients (**Figures 1D, E**). Our finding suggested that potential DNA methylated-related biomarkers were used to distinguish the different clusters of AML samples.

### Global Methylation Analysis in AML

**Figure 2:**
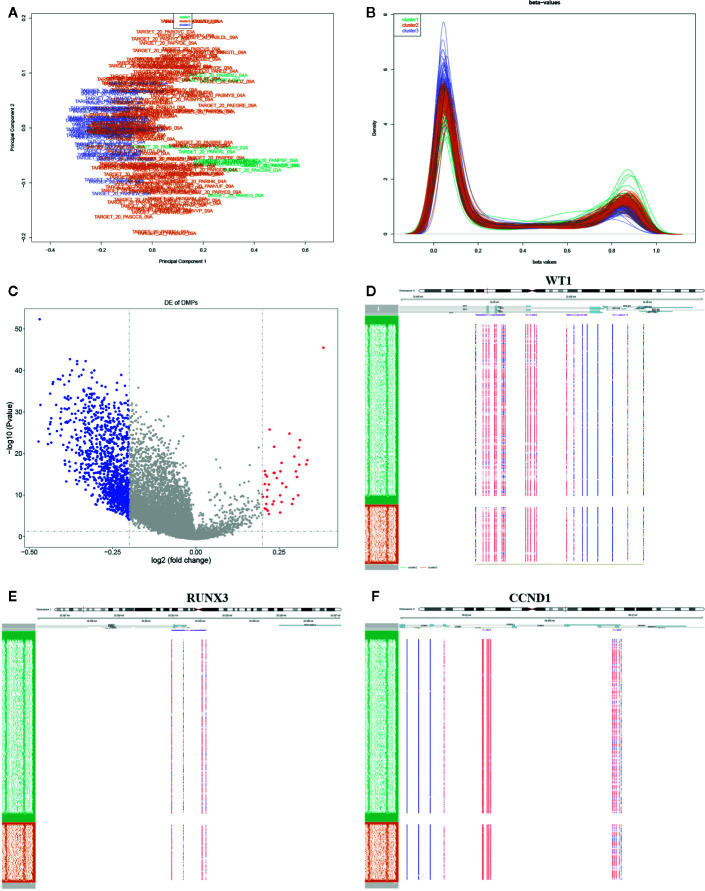
Global methylation analysis. **(A)** The principal component analysis (PCA) for the sample clustering. **(B)** The methylation density distribution plot showed the sample distribution trend and repeatability. **(C)** The volcano plot displayed the differential CpG sites between cluster 3 and cluster 2. **(D–F)** The methylated heatmap of the WT1, RUNX3, and CCND1.

The DNA methylation in three clusters was analyzed. Above all, the PCA results showed that the data among three clusters exhibited the distinct group-bias clustering and individual difference (**Figure 2A**), and the methylation distribution density plot showed the *β*-value DMPs were concentrated distribution at *β*-value <0.2 and *β*-value >0.8 (**Figure 2B**). A total 1,146 CpG sites were identified between cluster 3 and cluster 2, which included 40 hypermethylated and 1,106 hypomethylated CpG sites in AML (**Figure 2C**). Then, we analyzed the distribution of differentially methylated CpG sites in chromosomes; the results showed the differentially methylated CpG sites mainly were distributed in chromosomes 1, 2, 3, 7, 8, 9, 11, 15, 18, 22 (**Table 1**). We further explored the distribution of differentially methylated CpGs in function regions, including promoters, enhancer, TSS1500, TSS200, 1stExon, 5′UTR, 3′UTR (**Table 2**). Moreover, we assessed the distribution of differentially methylated CpGs in whole genome; we observed that low level of hypermethylated and high levels of hypomethylated CpGs in N-shore, S-shore, island, OpenSea, and 1,108 methylated genes were screened based on the DMPs in whole genome (**Table 2**). Next, we examined the distribution of differentially methylated CpGs in chromosomes; 2,856 DMRs were screened, and seven significant DMRs were filtered with a cutoff value no.cpgs ≥3, |meandiff| ≥0.2, Fisher (P value) >0.05 (**Table 3**). Importantly, seven significant DMRs included six hypomethylated CpGs (meandiff ≤ −0.2) and one hypermethylated CpGs (meandiff ≥ 0.2); a total 45 genes were involved in these regions, such as WT1, CCND1, RUNX3 (**Table 3**). Further, to analyze the DMRs in WT1, CCND1, RUNX3, we found that CpGs sites of WT1 were observed in the gene body at chromosome 11; the regions included N-shore, S-shore, and Island (**Figure 2C**). CpGs sites of RUNX3 were identified in the gene body, TSS1500, 1stExon, 5′UTR; the regions included OpenSea (**Figure 2D**). And the CpGs of CCND1 were observed in gene body, TSS1500, and 3′UTR (**Figures 2E, F**). Our results indicated there were obvious differentially methylated CpGs in AML.

**Table 1:** Top 20 differential DNA methylation sites analysis.

chr	pos	strand	Name	logFC (Δ*β*)	AveExpr	T	P Value	adj.P.Val
chr18	46064992	−	cg18530324	−0.468325207	0.787184384	−22.0824395	2.07E-57	5.18E-53
chr11	67205195	−	cg20792833	0.383999276	0.21481274	19.72927945	2.62E-50	3.28E-46
chr11	63754708	+	cg15564267	−0.377446517	0.73261242	−18.78234995	2.24E-47	1.87E-43
chr7	143013614	+	cg17922226	−0.356082336	0.736433242	−18.58115179	9.49E-47	5.38E-43
chr9	117567756	−	cg11809085	−0.330521921	0.653898995	−18.56380116	1.08E-46	5.38E-43
chr22	36561746	−	cg11835355	−0.356401904	0.753855936	−18.29816487	7.29E-46	3.04E-42
chr1	36273185	+	cg16019273	−0.3372841	0.77463379	−17.84615811	1.92E-44	6.86E-41
chr11	59633874	−	cg20018806	−0.304464436	0.732244749	−17.78020601	3.10E-44	9.51E-41
chr22	24032426	−	cg13253729	−0.371693501	0.794816667	−17.76643182	3.42E-44	9.51E-41
chr3	38347392	+	cg16558203	−0.311998205	0.759749132	−17.55934224	1.54E-43	3.86E-40
chr3	121773564	+	cg11874272	−0.223256244	0.635383699	−17.38944328	5.31E-43	1.11E-39
chr15	80264696	+	cg24924631	−0.342690612	0.80070758	−17.38857875	5.35E-43	1.11E-39
chr8	42397764	−	cg02641676	−0.377812598	0.678366575	−17.35460292	6.85E-43	1.32E-39
chr2	101618261	+	cg22809047	−0.398629676	0.744634703	−17.30520152	9.82E-43	1.75E-39
chr4	7046094	+	cg09424308	−0.35012	0.583291	−17.2282	1.72E-42	2.87E-39
chr18	46987532	+	cg12875426	−0.31187	0.707523	−17.1482	3.09E-42	4.83E-39
chr12	7343020	−	cg15754084	−0.41355	0.690298	−17.0269	7.49E-42	1.10E-38
chr19	50433949	−	cg04315771	−0.40662	0.644937	−16.9715	1.12E-41	1.56E-38
chr18	57566300	−	cg22549408	−0.38107	0.691926	−16.8211	3.38E-41	4.45E-38
chr1	1.61E+08	−	cg05659526	−0.42851	0.786543	−16.766	5.06E-41	6.33E-38

Chr, chromosome; pos, DNA methylation positions; strand, positive/negative chain; name, ID of DNA methylation positions; logFC, Δβ value; AveExpr, average of DNA methylation; P value, P value; adj. P.Val: adjusted P value.

**Table 2:** The number of hypomethylated and hypermethylated CpGs.

CpG islands	CpG NUM	hypermethylation	hypomethylation
N-Shore (0-2kb upstream of CpGs island)	241	4	237
S-Shore (0-2kb downstream of CpGs island)	219	7	212
Island (CpG islands)	345	4	341
OpenSea	308	24	284
N-Shelf (2-4kb upstream of CpG island)	21	1	20
S-Shelf (2-4kb downstream of CpG island)	12	0	12
Enhancer	156	5	151
Promoter associated	144	4	140
DNase-I-hypersensitive sites	169	6	163
TSS1500	505	10	495
TSS200	151	5	146
1stExon	278	9	269
5’UTR	264	7	257
3’UTR	14	3	11

CpG_NUM: CpG number.

**Table 3:** The regions of hypo- and hypermethylated CpG and genes.

chr	Regions	width	no. CpGs	HMFDR	Fisher	max diff	mean diff	overlapping. genes
chr11	32448769-32450692	1924	9	4.68E-28	2.89E-144	−0.442574744	−0.301686337	WT1
chr1	25290947-25292215	1269	4	7.81E-27	7.81E-87	−0.391155169	−0.328076988	snoU13, Y_RNA, SCARNA16, SCARNA21, U1, SCARNA17, SCARNA18, SNORD112, SNORA62, SNORA63, SNORD46, SNORA2, SNORD81, U3, SNORA51, SCARNA20, SNORA67, U6, SNORA70, SNORA77, SNORA26, U8, SCARNA11, RUNX3, SNORA31, SNORA42, SNORA40, SNORD64, ACA64, snoU109, SNORD60
chr8	41165699-41166738	1040	4	1.12E-35	1.46E-78	−0.436074868	−0.312695106	SNORA7, SFRP1
chr6	42927986-42928920	935	7	1.10E-26	2.16E-69	−0.304755388	−0.23534913	SNORA38, SNORA8, GNMT, SCARNA15, SNORA20
chr18	74961424-74964030	2607	7	2.74E-12	2.05E-54	−0.262945909	−0.213247912	GALR1
chr7	130125836-130126871	1036	6	1.80E-18	1.31E-42	−0.300512344	−0.208847136	MEST, RP11-2E11.10
chr11	69468789-69469290	502	5	3.16E-10	8.72E-33	0.292204442	0.230848843	CCND1, ORAOV1, SNORD43

### Identification of the DEGs in AML

**Figure 3:**
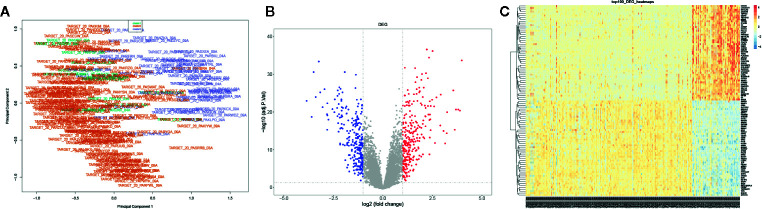
Identification of the DEGs in AML. **(A)** The PCA for the sample clustering. **(B)** The volcano plot displayed the DEGs between cluster 3 and cluster 2. **(C)** The heatmap exhibited the DEGs between cluster 3 and cluster 2.

We further identified the DEGs of AML samples: the mRNA expression profiles were grouped into three clusters based on the consensus clustering. The multi-dimensional scaling (MDS) results showed the clustering exhibited unbiased in the group; it indicated gene expression of samples was consistent and repeatable (**Figure 3A**). A total 529 DEGs were screened between cluster 3 and cluster 2 using Limma R package, including 270 upregulated DEGs and 259 downregulated DEGs with a threshold | log2 (fold change) | ≥1 and P-value ≤0.05 (**Figure 3B**). Besides, the top 100 genes containing 50 upregulated and 50 downregulated DEGs were identified using pheatmap R package (**Figure 3C**). Our data suggested that DEGs significantly distinguished between cluster 3 and cluster 2.

### Identification of the Biological Function of DEGs

**Figure 4:**
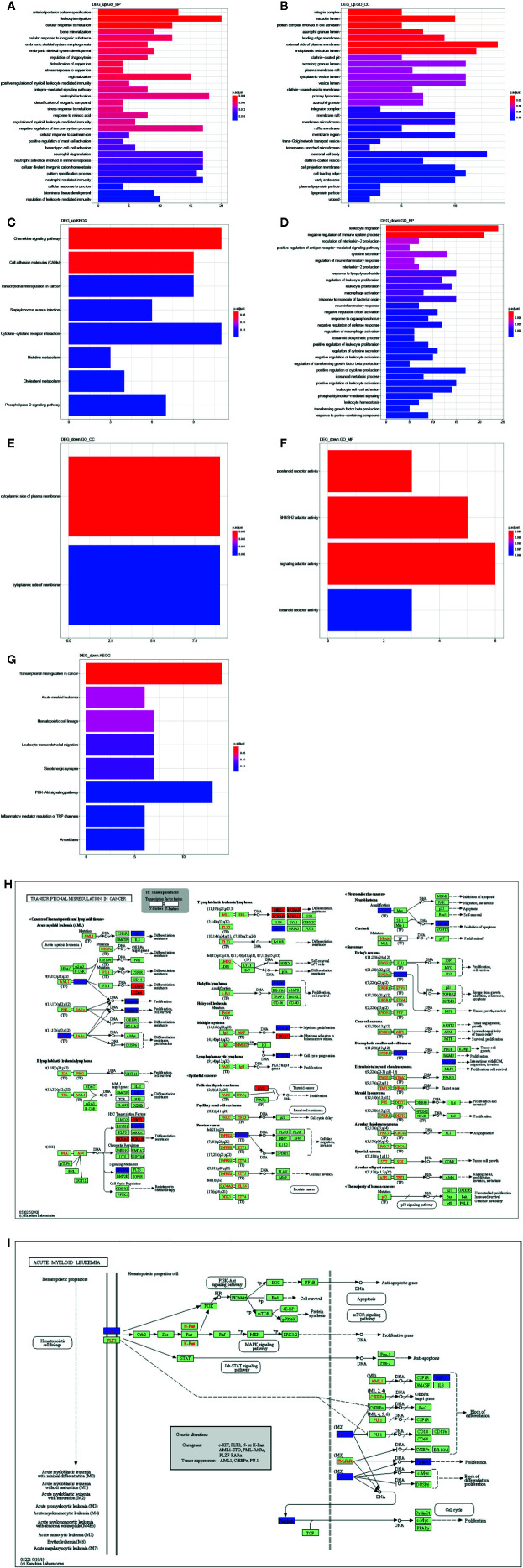
Function analysis of DEGs. GO annotation **(A)** and **(D)** BP, **(B)** and **(E)** CC, **(F)** MF, and **(C)** and **(G)** KEGG enrichment analysis the enriched pathways of upregulated/downregulated DEGs. **(H)** The transcriptional misregulation in cancer pathway (hsa052202) was enriched by KEGG analysis. **(I)** The acute myeloid leukemia pathways (hsa05221) were enriched by KEGG analysis.

We further investigated the biological function of DEGs by GO and KEGG pathway enrichment analysis using clusterProfiler R package. The biological processes of upregulated DEGs significantly enriched in AML related biological processes, such as leukocyte migration, positive regulation of myeloid leukocyte mediated immunity, regulation of myeloid leukocyte mediated immunity, regulation of leukocyte mediated immunity (**Figure 4A**), and cellular component enriched in integrin complex, vacuolar lumen. protein complex involved in cell adhesion, *etc.* (**Figure 4B**). Moreover, KEGG pathways were enriched in chemokine signaling pathway, cell adhesion molecules (CAMs), *etc.* (**Figure 4C**). Besides, the biological processes of downregulated DEGs were enriched in AML-associated biological processes, such as leukocyte migration, leukocyte proliferation, positive regulation of leukocyte proliferation, negative regulation of leukocyte activation (**Figure 4D**). Cellular component was enriched in the cytoplasmic side of plasma membrane and cytoplasmic side of membrane (**Figure 4E**). Molecular function was enriched in prostanoid receptor activity, SH3/SH2 adaptor activity, signaling adaptor activity, and icosanoid receptor activity (**Figure 4F**). And the KEGG pathways was enriched in transcriptional misregulation in cancer, acute myeloid leukemia, leukocyte transendothelial migration, *etc*. (**Figure 4G**). Taken together, the transcriptional misregulation in cancer and acute myeloid leukemia pathways might participate in AML progression; HOXA 10, HOXA9, HOXA11, RXRA, MEIS1, PBX3, ITGB7, ITGAM, FCGR1A, JUP, MPO, KIT, ZBTB16, RUNX1T1, CCNA1, SMAD1, TSPAN7, ERG, MYCN, WT1, CCND2, NTRK1, HPGD, PROM1 were involved in this pathway (**Figures 4H, I** **)**.

### Identification of the MDGs in AML

**Figure 5:**
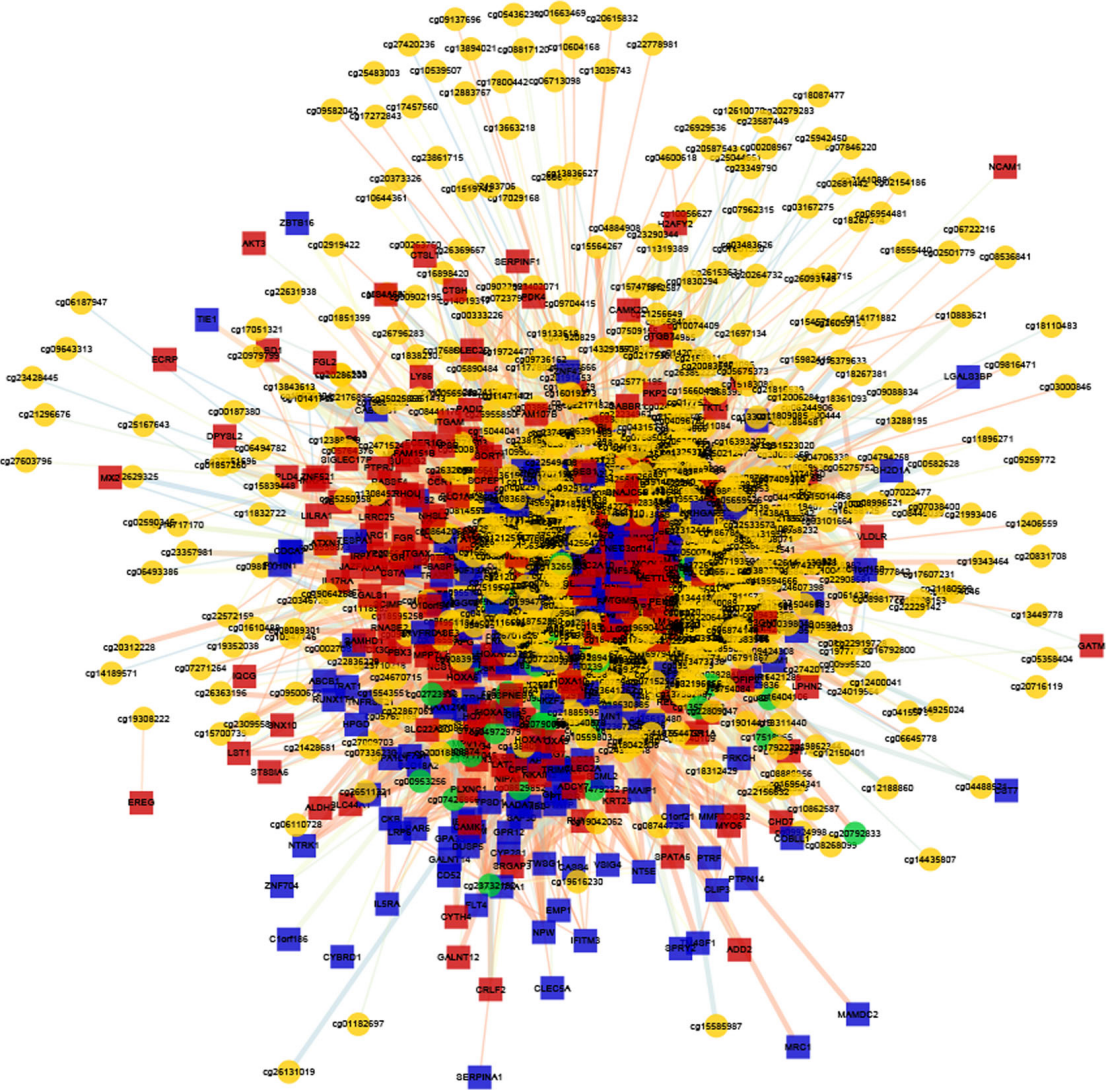
Correlation between DMG sites and DEG analysis. The co-expression network of DMGs and DEGs.

To determine the effects of DNA methylation on the gene expression, we examined the correlation between DMGs and DEGs by Pearson correlation analysis using psych R package. A total of 37,402 CpG-gene pairs were identified with a threshold |r| > 0.05 and adjusted P-value < 0.05, and there were 26,558 CpG-gene pairs which were related to downregulated genes (**Table 4**). The correlation between DMPs and DEGs was analyzed using STRING and visualized using Cytoscape (**Figure 5**). We found 18 DMGs containing 18 methylated regions in promoters, including three hypermethylated and 15 hypomethylated regions (**Table 5**). Moreover, correlation between 18 DMGs and AML was analyzed using varElect online tool; we found seven DMGs directly related to AML, and CD34, HOXA7, and CD96 exhibited the strongest correlation with AML (**Table 6**).

**Table 4:** Identification of DMGs and DEGs in AML.

Co-expression pattern	DEGs numbers	DMPs numbers	CpG-gene pairs
Hypermethylated downregulated genes	259	1,146	26,558
Hypomethylated upregulated genes	270	1,146	37,402

**Table 5:** Distribution of DMGs in different genomic locations.

DEGs	Ex	Cg sites	cor	p.adj	chr	Pos	Islands Name	Relation to Island	Gene Group	Methy type	logFC methy	adj. P. Val methy
CYB5R2	up	cg03826976	−0.531162252	6.67E-12	chr11	7695433	chr11:7694711-7695685	Island	TSS1500	Hypo	−0.382389921	1.96E-28
CAPG	up	cg10664272	−0.565735607	1.80E-14	chr2	85638053	chr2:85640969-85641259	N Shelf	TSS1500	Hypo	−0.262007243	2.93E-28
PARP3	up	cg12554573	−0.727270418	7.74E-32	chr3	51976667	chr3:51975612-51976505	S Shore	5′UTR, TSS1500, 1stExon, 1stExon, 5′UTR	Hypo	−0.231784355	5.54E-28
C2	up	cg14301635	−0.785164765	1.47E-41	chr6	31895203		OpenSea	TSS200, TSS200	Hypo	−0.336590739	5.96E-28
APOC2	up	cg27436184	−0.75568702	2.87E-36	chr19	45449006		OpenSea	TSS1500	Hypo	−0.295958592	4.02E-27
C3	up	cg17612991	−0.50948385	1.93E-10	chr19	6721016		OpenSea	TSS1500	Hypo	−0.274566124	4.80E-24
KCNC3	up	cg17838026	−0.596679336	4.82E-17	chr19	50832861	chr19:50833813-50834128	N Shore	TSS1500	Hypo	−0.385416053	2.84E-22
CLEC2A	up	cg27190239	−0.808757331	1.96E-46	chr12	10085174		OpenSea	TSS200	Hypo	−0.217426589	9.25E-22
CLEC2D	up	cg12810837	−0.590909294	1.53E-16	chr12	9822287		OpenSea	TSS200, TSS200	Hypo	−0.295239846	2.44E-21
GALM	up	cg05275752	−0.58856547	2.43E-16	chr2	38892887		OpenSea	TSS200	Hypo	−0.339937431	2.36E-18
PHACTR3	up	cg20674577	−0.500603373	7.16E-10	chr20	58179540	chr20:58179808-58180787	N Shore	TSS200	Hypo	−0.270475464	3.57E-18
PLD3	up	cg20513206	−0.514883287	8.56E-11	chr19	40855502	chr19:40854180-40854733	S Shore	5′UTR, 5′UTR, TSS1500	Hypo	−0.246633135	1.51E-17
LIN7A	up	cg05647859	−0.553169576	1.68E-13	chr12	81331718	chr12:81330608-81331514	S Shore	TSS200	Hypo	−0.33747097	1.02E-14
EREG	up	cg19308222	−0.524719892	1.86E-11	chr4	75230391		OpenSea	TSS1500	Hypo	−0.247715322	4.81E-11
HOXA7	up	cg26511321	−0.50856622	2.22E-10	chr7	27196790	chr7:27198182-27198514	N Shore	TSS1500	Hypo	−0.209905127	3.94E-10
CD96	down	cg04039397	−0.857524793	4.28E-59	chr3	111260783		OpenSea	TSS200;TSS200	Hyper	0.309673775	3.14E-22
ZG16B	down	cg05461841	−0.703756457	1.37E-28	chr16	2879944		OpenSea	TSS1500	Hyper	0.201967332	2.16E-10
CD34	down	cg03583857	−0.808135071	2.58E-46	chr1	208085022	chr1:208084098-208084513	S Shore	TSS1500;TSS1500	Hyper	0.231254816	1.33E-08

**Table 6:** Correlation of DMGs and AML analysis.

Gene Symbol	Marched Phenotypes	Score	Log10 (P)	Average Disease Causing Likelihood
CD34	acute myelogenous leukemia, acute myeloid leukemia	58.62	2.39	42.0
HOXA7	acute myelogenous leukemia, acute myeloid leukemia	44.86	2.10	43.1
CD96	acute myelogenous leukemia, acute myeloid leukemia	40.62	1.95	57.4
C3	acute myelogenous leukemia, acute myeloid leukemia	7.76	1.50	67.8
PARP3	acute myeloid leukemia	5.21	1.40	19.8
PLD3	acute myeloid leukemia	1.09	1.09	69.8
EREG	acute myeloid leukemia	0.07	0.53	67.0

### Construction of Three-MEGs Clinical Prognostic Biomarkers for AML

**Figure 6:**
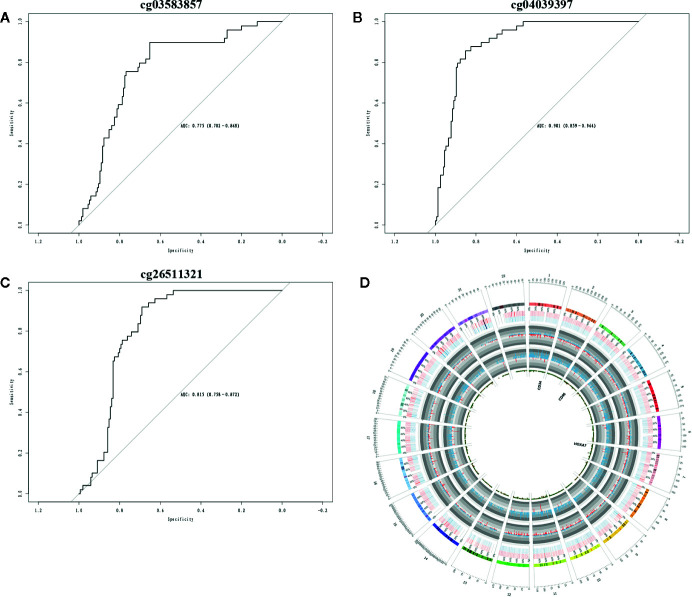
Clinical prognostic biomarker analysis. **(A–C)** The ROC plots of CD34, CD96, and HOXA7 for the consensus clusters. **(D)** The circular plot of CpGs.

The Cox regression analysis was used to explore the role of methylation on AML. The clinical significance of three CpG sites (cg03583857, cg26511321, cg04039397) of candidate methylated genes was investigated by Cox regression analysis. The results showed AUC values of cg03583857 is 0.775 (**Figure 6A**), AUC value of cg04039397 is 0.901 (**Figure 6B**), and AUC value of cg26511321 is 0.815 (**Figure 6C**). The methylated regions were illustrated in **Figure 6D**. Therefore, three CpG sites cg03583857, cg26511321, cg04039397 of CD34, HOXA7, and CD96 might be use as the clinical prognostic biomarkers.

## Discussion

The overall survival of pediatric AML has increased in recent years with the development of chemotherapy, supportive care, and savage therapy (4); the overall survival increased from 49 ± 3% to 76 ± 4% between 1987 and 2012 (5). However, chemotherapy alone is the main treatment. Some recurrent and refractory AMLs are insensitive to chemotherapeutic drugs, which seriously restrict the improvement of survival rate in patients with AML. It has been reported that epigenetic aberrations are important reasons of leukemogenesis (17), such as the DNA methylated miR-150-5p which is mediated by DNA methyltransferase 1 that promotes the FGFR1-drived leukemogenesis (25). DNA methylation plays an important role in the regulation of the occurrence and development of AML (26). Some scholars have added DNA methylation inhibitors to refractory AML patients and found that the vast majority of AML patients can significantly prolong the survival time (24). Besides, the methylated GGH promoter associates with the variability of methotrexate responses in AML patients (27). Methotrexate as a first-line chemotherapy drug for clinical treatment of AML; DNA methylation can reduce the response of the human body to methotrexate and then directly affect the therapeutic effect. Inhibition of DNA methylation can increase the sensitivity of the human body to methotrexate (28). Clinical studies have found that the combined use of anti-DNA methylation drugs can significantly improve the effect of chemotherapy and prolong survival time (22).

In this study, we clustered AML samples into three consensus clusters at k = 3, and the DMGs were identified and validated in AML, including CD34, HOXA7, and CD96. CD34 plays as a role in response to iodine-131 radiotherapy in thyroid carcinoma (29). HOXA7 is a member of the HOX family which was involved in the development of multiple cancer types (30). CD96 has been reported as a novel immune checkpoint in glioma and associates with immune cell infiltration (31). High expression of CD34 and DNA methylation has been found in children with neuroepithelial tumors. Further studies have found that there are frequent genetic abnormalities involving B-Raf proto-oncogene (BRAF) or fibroblast growth factor receptors 2 and 3 (FGFR2, FGFR3), which can induce the occurrence and development of neuroepithelial tumors (32). In a methylation study on the early diagnosis of non-small cell lung cancer, it has found that the expression of HOXA7 methylation had high sensitivity and specificity in the diagnosis of non-small cell lung cancer (33). The low expression of HOXA7 methylation also exists in breast cancer cells, while the normal expression of HOXA7 methylation in normal tissues adjacent to breast cancer, ndicating that HOXA methylation expression plays an important role in the occurrence and development of breast cancer (33). CD96 is related to TIGIT, CD226, CRTAM, TIM-3, PD-L1, CTLA-4, and STAT3. These checkpoint proteins have additive antitumor effects, and CD96 plays an important role in immune response (34); azacytidine and decitabine have become inhibitors of DNA methylation combined with chemotherapeutic drugs, which can prolong the survival time of AML patients and improve the prognosis of AML patients (22).

Three key targets of DNA methylation on AML disease from multiple angles, which can provide genetic theoretical evidence for the early diagnosis of AML were further analyzed to provide clinical evidence for the inhibition of DNA methylation to improve the therapeutic effect and to provide ideas for the diagnosis and treatment of refractory and recurrent AML. It provides hope for AML patients to prolong their survival time and enter the clinical remission stage as soon as possible.

Here, we performed an integrated analysis of DNA methylation and gene expression; the abnormal expression genes involved in AML were identified. Moreover, our finding suggested that DNA methylated promoter of genes showed low mRNA expression, which is related to the overall survival of AML patients in cluster 3 which is significantly shorter than AML patients in cluster 2. Hypermethylation of CD34 and CD96 at cg03583857 and cg04039397 significantly inhibited mRNA expression, and hypomethylation of HOXA7 at cg26511321 obviously accelerated mRNA expression. CD34 is one of the members of the cluster of differentiation 3 (CD34) family, which is a type-I transmembrane sialomucins and acts as the marker of hematopoietic stem cells and vascular (35). In gastrointestinal stromal tumor (GIST) patients, a strong inverse correlation between DNA methylation degree and CD34 expression has been evaluated by immunohistochemical staining. The expression of CD34 has been modulated by DNA methylation in a site-dependent manner in GISTs.

Abnormal expression of HOX gene is a common feature in AML (36). However, the molecular mechanism of HOX gene in AML remains unclear. Previous study demonstrated that CCCTC binding factor (CTCF) mediated aberrant expression of HOX in AML. Di Vinci, A. et al. have shown that HOXA7 is a methylation target associated with aggressive behavior in meningiomas. The above studies have confirmed that the methylation of HOXA7 is closely related to the occurrence and development of tumors.

CD96 is an immune related gene which acts as an immune related marker in AML (34), high expression of CD96 associated with poor prognosis in *de novo* AML (37). It has been indicated that CD96 is regulated by differential DNA methylation to change the mRNA and protein expression. The above studies proved that CD96 methylation interferes its mRNA expression, which indirectly affects the occurrence and metastasis of cancer.

In summary, we performed an integrative analysis of multi-omics in AML and identified the differential DNA methylated genes such as CD34, HOXA7, and CD96 and explored DNA methylation and clinical implication of CD34, HOXA7, and CD96 in AML.

## Data Availability Statement

Publicly available datasets were analyzed in this study. This data can be found here: https://ocg.cancer.gov/programs/target/data-matrix.

## Author Contributions

TZ and JZ contributed to the conception of the study. YX and CC contributed significantly to analysis and manuscript preparation. JZ performed the data analyses and wrote the manuscript. WG, CZ, XC, LG, CC, and YX helped perform the analysis with constructive discussions. All authors contributed to the article and approved the submitted version.

## Funding

This work was supported by National Natural Science Foundation of China [Grant number 81771589], the Key Project of Tianjin Health Care Professionals [Grant number 16KG166] and the Program of Tianjin Science and Technology Plan [Grant number 18ZXDBSY00170].

## Conflict of Interest

The authors declare that the research was conducted in the absence of any commercial or financial relationships that could be construed as a potential conflict of interest.

## Supplementary Material

Supplementary Figure 1 Unsupervised clustering analysis in AML. **(A)** The CM plot exhibited the clusters at k = 1, 2, 4, 5, 6. **(B)** The Tracking plot indicated the consensus cluster of items at each k. **(C)** The cluster-consensus plot showed the consensus value of each k. **(D)** The item-consensus plot exhibited the multiple item-consensus values for an item at the k clusters.
