# Supplementary figures and images for: Integrative Analysis of Multi-Omics Identified the Prognostic Biomarkers in Acute Myelogenous Leukemia
Source: Front Oncol. 2020 Dec 10;10:591937. doi: 10.3389/fonc.2020.591937 (PMC7758482; doi:10.3389/fonc.2020.591937)

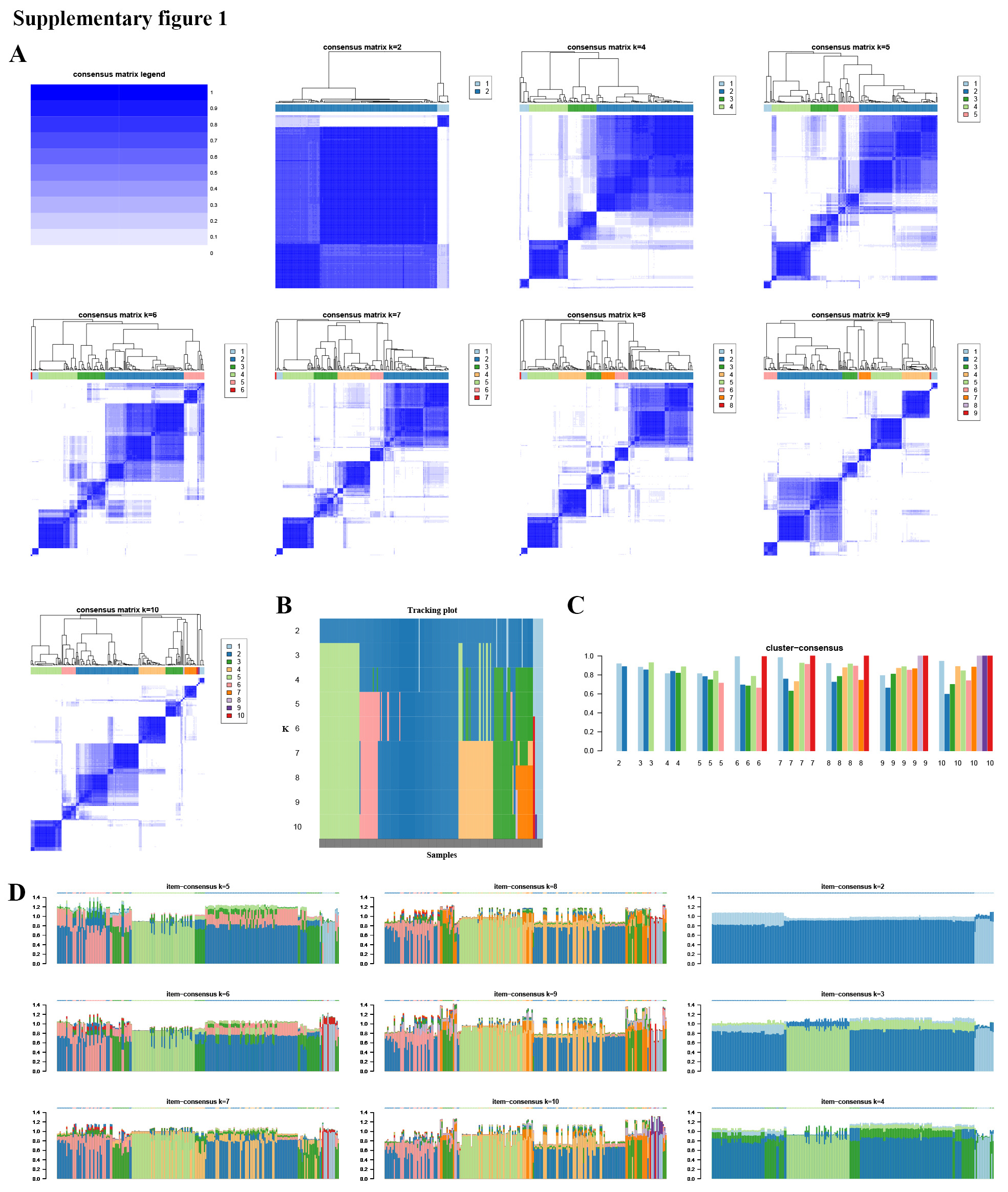

Supplement: Supplementary Figure 1 — Unsupervised clustering analysis in AML. (A) The CM plot exhibited the clusters at k = 1, 2, 4, 5, 6. (B) The Tracking plot indicated the consensus cluster of items at each k. (C) The cluster-consensus plot showed the consensus value of each k. (D) The item-consensus plot exhibited the multiple item-consensus values for an item at the k clusters. [file Image_1.jpeg]
